# Professional perspectives on impacts, benefits and disadvantages of changes made to community continence services during the COVID-19 pandemic: findings from the EPICCC-19 national survey

**DOI:** 10.1186/s12913-022-08163-3

**Published:** 2022-06-15

**Authors:** Cecily Palmer, Davina Richardson, Juliette Rayner, Marcus J. Drake, Nikki Cotterill

**Affiliations:** 1 National Institute for Health Research Applied Research Collaboration West (NIHR ARC West), University Hospitals Bristol and Weston NHS Foundation Trust, Bristol, UK; 2 Bladder & Bowel UK, Disabled Living, Burrows House, 10 Priestley Rd, Wardley Industrial Estate, M28 2LY Manchester, UK; 3 ERIC, The Children’s Bowel & Bladder Charity, 36 Old School House, Kingswood Foundation, Britannia Rd, BS15 8DB Bristol, UK; 4 Translational Health Sciences, Bristol Medical School, Bristol Urological Institute, Southmead Hospital, Bristol, BS10 5NB UK; 5 Faculty of Health and Applied Sciences, School of Health and Social Wellbeing, University of the West of England, Blackberry Hill, Bristol, BS16 1DD UK

**Keywords:** Incontinence, Community continence services, COVID-19, Impact, Remote consultations, Remote appointments, Digital health, Redeployment, Access to care, Qualitative

## Abstract

**Background:**

The COVID-19 pandemic required changes to the organisation and delivery of NHS community continence services which assess and treat adults and children experiencing bladder and bowel difficulties. Although strong evidence exists for the physical and mental health benefits, improved quality of life, and health service efficiencies resulting from optimally organised community-based continence services, recent audits identified pre-pandemic pressures on these services. The aim of this study was to explore professional perceptions of changes made to community continence services due to the COVID-19 pandemic and consequent impacts on practice, care provision and patient experience.

**Methods:**

Online survey of 65 community continence services in England. Thematic analysis using constant comparison of open-ended questions. Frequency counts of closed-ended questions.

**Results:**

Sixty-five services across 34 Sustainability and Transformation Partnership areas responded to the survey. Use of remote/virtual consultations enabled continuation of continence care but aspects of ‘usual’ assessment (examinations, tests) could not be completed within a remote assessment, requiring professionals to decide which patients needed subsequent in-person appointments. Remote appointments could increase service capacity due to their time efficiency, were favoured by some patients for their convenience, and could increase access to care for others. However, the limited ability to complete aspects of usual assessment raised concerns that diagnoses could be missed, or inappropriate care initiated. The format also restricted opportunities to identify non-verbal cues that could inform professional interpretation; and made building a therapeutic relationship between professional and patient more challenging. Remote appointments also posed access challenges for some patient groups. A third of participating services had experienced staff redeployment, resulting in long wait times and some patients being left without care; or reported additional caseload, which had delayed care provision for patients with continence issues. Participants perceived continence care to have been deprioritised, and more generally undervalued, and called for greater recognition of the impact of continence care.

**Conclusions:**

Remote appointments offer efficiency and convenience. However, ‘in-person’ approaches are highly valued for optimum quality, patient-centred continence care, and good team relationships. Failure to restore redeployed continence staff will diminish patient health and quality of life, with associated costs to the NHS.

**Supplementary Information:**

The online version contains supplementary material available at 10.1186/s12913-022-08163-3.

## Background

The COVID-19 pandemic necessitated rapid changes to the UK healthcare system to limit infection spread and release resources to manage cases of COVID-19 [1]. Across NHS acute and community services, changes were made to care organisation and delivery including staff redeployment to ‘priority’ services, prioritisation of ‘essential’ care, and discharge of medically fit patients into the community [1–3]. A key change was the rapid implementation of telephone and video facilitated remote consultations [4, 5] which minimised physical contact between patients and professionals, although a strong policy imperative to make use of digital technologies within health and social care was already in place [6].

A small number of studies have examined the impact of the COVID-19 pandemic on NHS services, primarily in acute care [1, 2]; few have focussed on community-based services. Studies of the impact of changes made in response to the pandemic on practice, access to, and quality of care are needed to inform future delivery and offer opportunity to explore potential consequences of widespread uptake of digitally enabled ‘remote’ ways of working in health care settings [5, 7].

NHS community continence services assess and treat adults and children experiencing bladder and/or bowel difficulties. In addition they provide training and education to healthcare practitioners and carers. Incontinence problems (urinary and faecal) are extremely common and affect people of all ages [8, 9]. One in 10 UK children are estimated to experience difficulties [10]. Urinary incontinence affects 25-45% of women, and 11-34% of men [8, 11] while prevalence of faecal incontinence is between 9 [8] and 11% [11] in the adult population. Incontinence problems are most common amongst older adults in long-term care [12]. They are known to impart a heavy social and emotional burden; severely affecting quality of life for those affected and their carers [8, 13–16].

Despite its prevalence and impact, incontinence is a treatable condition [15]; a significant proportion of difficulties can be cured or improved with simple and non-invasive interventions delivered in the community [8, 15, 17, 18]. National guidelines outline the essential role in the NHS of integrated and comprehensive community-based continence services, led by specialist continence nurses providing evidence-based care for patients and for which ‘cure rather than containment should be the principle aim of treatment’ [15, 19]. The early and effective treatment of continence difficulties in children can alleviate potential negative impacts on self-esteem and social behaviour and may prevent the continuation of difficulties into adolescence and adulthood [20, 21], thereby improving quality of life for young people and their families [10]. Amongst adult or older populations, good continence care may avoid subsequent health impacts such as urinary tract infection, pressure ulcers and falls, thereby reducing emergency admissions and lengthy hospital stays associated with them [22]. Associated mental health declines due to isolation and depression may also be averted [23]. First line conservative strategies can avoid unnecessary treatment in acute care, reduce pad and product use, and improve quality of life, with associated cost savings [8, 13, 22].

Recent audits have identified pressures on community continence services [8, 19], and the recent pelvic floor report highlighted that these services have ‘long been at the back of the queue for funding and prioritisation’ [24]. The COVID-19 pandemic is likely to have added to these issues. This paper reports findings from a national survey of continence care professionals (EPICCC-19) which aimed to determine changes made to community continence services in response to the COVID-19 pandemic and explore perceptions of impact on practice, care provision and patient experience.

## Methods

### Sample and data collection

The study used a cross sectional survey design to explore practice across a broad sample of services, with mixed qualitative and quantitative methods. Between 01/12/2020 and 10/06/2021, 142 continence and/or bladder and bowel services providing care for adults and children in the community in England were invited to participate in an online survey exploring the impact of the COVID-19 pandemic on continence care provision; no exclusion criteria were applied. An email invitation and Participant Information Sheet was sent to the service email address or named lead at the service which provided information about the study and included an electronic link to the online survey [25]. Study team contact details were included to allow potential participants to ask questions. Services were invited from all 44 Sustainability and Transformation Plan (STP) footprint [26] areas in England in two phases; 101 in December 2020, and 41 in April 2021 (once contact email located). Those who had not completed the survey were sent up to four email reminders at monthly intervals.

Survey questions were developed in collaboration with continence care professionals following an earlier pilot in a single Clinical Commissioning Group (CCG) area. The online survey required completion of an initial consent process before progression to 14 survey questions. 11 questions invited open ended responses in a free-text format, three were closed ended [see Additional file 1]. The survey concluded with an open comments section. Use of a survey design with open ended questions allowed participants scope to report perceptions and experiences without restriction to predetermined categories, while also maximising recruitment of a broad sample of services. Survey responses were saved into the online survey platform and downloaded at intervals during data collection.

### Patient and Public Involvement (PPI)

An earlier survey draft was developed in partnership with the Bladder and Bowel Confidence Health Integration Team PPI group [27], to ensure public and patient perspectives relating to impacts of importance informed survey design even though the survey was designed to capture the service provider perspective.

### Data analysis

Survey data comprising responses provided by 41 services was downloaded in February 2021 to develop a preliminary coding framework for the 11 open-ended questions. Firstly, frequency counts of ‘affirmative’/ ‘negative’ responses to open-ended questions were (where possible) undertaken e.g., of responses to Q4a the number that perceived ‘benefits’ and ‘no benefits’ to changes made to services [see Additional file 1]. Open-ended question responses were then analysed using thematic analysis and the technique of constant comparison [28]. Responses were closely and repeatedly read and topics relevant to the question topic were deductively coded and compared. Emerging categories were summarised in a preliminary descriptive framework for each open-ended survey question [29], with the most common topics presented first.

To increase reliability and validity the study team shared coding of response data for a subset of questions and met to discuss preliminary categories. Response data that was not deductively coded as relevant to the specific survey question was coded inductively. Inductively coded topics were compared horizontally (across all questions) which identified four emergent categories of importance; ‘staff redeployment’, ‘service relationship with community nursing’, ‘service relationship with care/residential homes’ and ‘continence care downgraded’. These topics were coded and compared across all survey response data to further analyse their importance.

Response data from the final 24 services was downloaded in June 2021. Open-ended questions were analysed using the preliminary coding framework which was extended/modified where necessary and coded horizontally for the inductively identified topics. Final descriptive summaries for each question were discussed at a team meeting and key topics recurrent across the survey questions identified and labelled as themes. In addition, frequency counts were generated by answer category for the three closed questions and response frequency counts finalised for all questions. Responses to Q3 [see Additional file 1] were used to identify the geographical spread of survey responses.

This paper presents themes identified from thematic analysis of data from 11 open-ended survey questions, presented under four headings; Changes to adult and paediatric community continence services, Perceived benefits and disadvantages of changes, Patient and carer response to service changes; and Future service provision. Anonymised data extracts are provided for each theme (see also Additional file 2). Findings of closed ended questions are included for completeness.

### Ethics

This study was approved by the University of Bristol Faculty of Health Science Research Ethics Committee [Ref: 111563] and informed consent received from all participants via an electronic consent process within the online survey.

## Results

### Participant sample

65 participants representing adult (40), paediatric (13), joint (10) or other (2) community continence services completed the survey (Q2). Geographical area of service provision (Q3) linked to STP area identified that 52 services from 34 STP areas (N-44) had responded to the survey. Geographical spread of responses may be higher than this, as STP area for 13 participating services could not be identified (see Table 1: Q3—Geographical spread of service responses).

**Table 1:** Q3 Geographical spread of service responses

Region	No. of STP areas in region	No. of services invited to survey	Responding services: provided geographical information linkable to STP area	No. of STP areas from which service response received	Responding services: provided geographical information at regional level only	Responding services: provided no geographical information
North East & Yorkshire	5	18	6	4/5 STPs	1	
North West	4	18	5	2/4 STPs	-	
Midlands	11	22	7	6/11 STPs	2	
East of England	6	18	6	5/6 STPs	1	
London	5	32	11	5/5 STPs	1	
South West	7	16	8	7/7 STPs	3	
South East	6	19	9	5/6 STPs	-	
**TOTAL**	**44 STP areas**	**142**	**52**	**34 STP areas**	**8**	**5**

### Changes to adult and paediatric community continence services

#### From in-person to remote care provision

Sixty-four of sixty-five participants reported changes made to the way in which their service provided care due to the ongoing COVID-19 pandemic (Q4). Unsurprisingly, ‘in-person’ (face-to-face) appointments had been largely replaced by remote/virtual consultations facilitated by telephone/video call. Rapid adoption of remote appointments and working practices allowed services to continue to provide care, support and advice during the pandemic. However, they also limited professionals’ ability to fully assess patients and required clinical decisions to be made about patients’ need for a physical examination or diagnostic test, where previously these elements would be a routine part of an in-person assessment.

4840 ‘Reduced face to face appointments meant that we were not able to provide the usual care i.e. physical examinations at the initial appointment’

Those deemed to require examination or tests would be placed on a waiting list for the outstanding part of the continence assessment to take place in a subsequent in-person appointment.

4588 ‘All 1st assessments by phone - the clinician makes the clinical decision to see in clinic or HV (home visit) for physical examination’

Thirty-one participants reported continued provision of some ‘in-person’ appointments (home visits or in clinic). However, this provision was described as far less than ‘usual’ (‘very limited’ or ‘minimal’) and many described that in-person appointments were given to ‘those that really need’ or for ‘essential’ or ‘emergency’ needs; commonly specified as those requiring physical examinations, diagnostic investigations, or catheter care although services reported varied criteria.

7663 ‘… face to face appointments only if examination is required or the patient has communication or cognitive impairment’

Provision of ‘in-person’ appointments varied at the time of participation in the study (Dec 20-June 21); some participants reported in-person clinics had been reinstated, others that they had restarted and then ceased. Two participants representing paediatric services reported that no in-person appointments were currently provided.

4265 ‘Clinics stopped in 1st lockdown but up and running since’

7189 ‘Currently not seeing clients face to face’

#### Redeployment, service suspension, additional responsibilities

Twenty participants reported that continence staff had been redeployed (commonly to community/district nursing [CN/DN] teams) and the continence service had been partially closed/suspended. Consequently, these services had been reduced to providing urgent care only, while ‘routine’ continence care was put on hold and clinics closed. Some services had stopped accepting new referrals for a period; others continued to take referrals but patients requiring specialist help or needing an in-person appointment had been placed on a waiting list until the suspended aspects of the service were reinstated.

4840 ‘Staff were deployed into the community teams and unless urgent, all routine continence care was put on hold’

Six participants (distinct from those reporting redeployment to CN/DN teams) reported that some patients usually seen by the community/district nursing teams had been added to the continence team caseload to reduce the CN/DN workload. The continence teams managed the additional responsibilities, which included supporting with catheter care, bladder scans, basic continence assessments, or continence assessments for housebound patients, in addition to their usual continence work.

4588 ‘All continence referrals for a basic assessment were directed into the [continence] ...team...to reduce work load on DN teams’

#### Access to residential and care homes

Continuation of continence care delivery in nursing/residential homes varied; some participants reported no access due to the need to protect vulnerable residents. Participants undertook assessments by telephone with care home staff and relied on staff to provide skin condition reports and completed bladder and bowel diaries for patients in their care.

#### Remote working, training and educational activities

In addition to remote care delivery, continence teams undertook meetings, clinical supervision and peer support remotely. Additionally, some services delivered clinical training and/or patient education activities remotely and increased electronic provision of information. For other services clinical training opportunities had reduced due to restrictions on gatherings or had stopped all together.

1194 ‘Toilet training education sessions for parents completed via zoom set up by schools’

### Perceived benefits and disadvantages of changes to community continence services

Participants reported benefits and drawbacks to the changed way in which their services provided continence care (Q4,4a,4b) and described impacts for care and patient experience (Q8, Q9, Q9a, Q10, Q10a, Q16).

#### Remote care provision—efficiencies and limitations

Use of remote appointments was widely reported to have increased service capacity due to their time efficiency in contrast to in-person appointments which often entailed travel time between clinics, to home visits and/or schools. Remote appointments allowed more patients to be ‘seen’ in the time available, and for advice/interventions to be started more promptly.

6777 ‘Ability to assess patients more quickly and in greater numbers as the virtual clinics are more time efficient than face to face’

Some services, therefore, reported that the remote completion of initial and/or review assessments had positively affected waiting times for in-person appointments.

2614 ‘reduce[d] waiting list time for clinic appointments as initial assessments were commenced via telephone consultations.’

However, participants also emphasised the limitations of remote appointments in contrast to in-person interactions between professional and patient. Assessments undertaken remotely were described as fundamentally ‘incomplete’ due to inability to undertake physical examinations and some diagnostic tests.

8186 ‘Assessment has limitations and relies on the nurses skill and knowledge far more now, as they have to decide if a scan or examination is needed rather than everyone getting offered this as routine’

Participants perceived that an increase in pressure sores would result from being unable to do skin checks for patients and that inevitably some diagnoses would be missed or inappropriate treatment started.

2018 ‘Last year [the service] had 36 ... pressure sores identified during face-to-face assessment. None during the 6-month telephone assessments. Concern pressure sores will have been missed’

For some services, inability to complete assessments in remote appointments had increased waiting lists for the ‘in-person’ appointments that were necessary so that examinations/tests could be completed. This was further intensified by staff shortages (due to isolation or shielding) and restricted numbers of available appointments due to clinic cancellation, restrictions on social groupings, and increased cleaning time between patients.

5114 ‘Unable to complete physical assessment and diagnostics. Patients needing follow up …can impact on the waiting list’

Remote appointments were also described as ‘less effective’ than in-person ones because they limited opportunities to pick up on non-verbal cues or subtle holistic signs, related to both patient and their physical/social environment, that would have informed professionals’ interpretation had the interaction taken place face-to-face.

4281 ‘We need to be mindful to the less obvious cues when engaging in telephone consultations, such as safeguarding and domestic abuse’

Concerns were expressed about using remote appointments for vulnerable children, for whom neglect may be part of their continence problem, and for children with safeguarding needs and at risk of domestic abuse. This is because the format limited ability to closely consider treatment adherence and to evaluate general and non-verbal cues. Further, remote appointments often increased reliance on information from ‘others’, such as parents or care staff, from which professionals had to make diagnoses or plan care.

7189 ‘We are more reliant on parents perception on their child's continence issue’

In addition, participants perceived that lack of in-person contact undermined the building of a therapeutic relationship between patient and professional. Rapport, closeness, and touch were prevented by remote consultations, which were considered important for discussion and assessment for an issue as personal as continence care:

3233 ‘[Drawbacks are] not being able to use touch as a communication aid, formality and relative anonymity’

Many participants perceived in-person appointments to be the gold standard for engaging patients and providing high quality continence care, and that the standard of care was potentially lessened by remote service provision.

2984 ‘there is a general feeling that assessments were incomplete therefore care given is less robust than it should have been’

9792 ‘As good as telephone and video appointments can be, it will never replace face to face contact... I feel it also helps build the relationship between clinician and patient better’

#### Remote care provision—convenience and inaccessibility

Participants commonly reported better patient attendance at remote appointments and fewer ‘did not attends’ (DNA). Remote consultations were perceived to be more convenient for (some) patients, avoiding school/work disruption, time-consuming or costly travel to an appointment, and preferable to many due to reduced risk of exposure to COVID-19.

1194 ‘Families seem to like telephone appointments for convenience and not needing to travel’

Some participants perceived potential for increased access to care for those who had difficulty accessing a clinic venue in person, as they would be able to access care through virtual means and receive specialist input normally requiring an ‘in-person’ visit.

2793 ‘those with reduced mobility, anxiety and depression and other conditions … can still access the service through virtual and telephone means’

Some participants (representing both adult and paediatric services) perceived remote appointments were less embarrassing or intimidating for some patients and facilitated greater ease of conversation.

3361 ‘Patients have stated that the initial phone call is less embarrassing than a face to face consultation’

However, participants also described access and ease of use challenges surrounding remote appointments, specifically for patients (and carers) with a communication difficulty or for whom English is not a first language, and for patients with a learning disability, mental health difficulty, cognitive/sensory impairment, or hearing/visual difficulty, vulnerable people and those who live alone and without someone to advocate for them.

4281 ‘[for] patients with a learning disability; cognitive impairment or hearing difficulty telephone contact can be challenging despite a carer being available’

Participants reported it was common for older patients (as well as some others) to have no access to the technology (internet, smartphone, computer) required for a video consultation, or if available to them, to be unfamiliar with using it.

7749 ‘Some cannot afford internet or are not familiar with the technology used’

Telephone calls were described as the main way in which older people were contacted remotely. Written information leaflets emailed in place of verbal explanation were inaccessible to those with limited literacy.

Participants expressed discomfort that some patients (children, those in care homes) could be ‘excluded’ from their assessment as a family member/carer took over a consultation due to the remote format being less accessible for the patient themselves, effectively bypassing the patient from their own care.

8186 ‘Carers have the most up to date information, but this cuts out the patient’.

Their use was also perceived to disadvantage children and adults with complex or additional needs due to the longer time taken to build rapport and develop an appropriate care plan, than when an in-person approach was used.

9792 ‘...children with complex needs, or those who require more individualised care - it is hard to get to know patients for the first time through video appointments… it can take a while to form an appropriate care plan’

Participants also identified that lack of privacy in the home could restrict people from disclosing their problems during a remote consultation.

3233 ‘the fact that other family members are also at home at time of call, led to patients not fully expressing their problems’

#### Impact of redeployment and additional responsibilities

Participants representing services that had been partially suspended, due to redeployment, described increased waiting times for assessment and treatment, leading to patients potentially experiencing worsening continence problems for longer. Several emphasised that some patients were receiving no care due to the reduced continence service and/or community nursing ceasing some continence work.

0896 ‘Our service has developed long waiting lists’

9422 ‘Very little face to face assessments. No skin checks, no pad fitting checks and no treatment plans’

Some services that had gained referrals usually cared for by other teams found the increased caseload reduced their capacity to provide care to patients with continence needs. This delayed care for these patients, although some staff worked unpaid overtime to manage the increased workload.

4265 ‘Continence care staff used to support community nursing so continence patients care sometimes delayed’

The impact of appointment backlogs on staff morale was also described:

4223 ‘backlog of clinic appointments creating a very long waiting time which can be overwhelming for staff’

Some participants representing redeployed services and those given additional responsibilities did, however, describe benefits resulting from close working with community nursing teams including increased understanding, productive relationships and reciprocal skills sharing between continence teams and community teams.

7126 ‘staff worked with neighbourhood nursing teams and improved links and gave training’

#### Continence care downgraded and undervalued

Participants reported that delayed assessment, increased waiting lists and restrictions on in-person appointments had led to a downgrading of continence care to pad supply, with little focussed effort being made to support the achievement of continence.

8821 ‘continence assessments were delayed by months and more often than not became a pad assessment’

For many, the redeployment of their service staff or addition of community nursing responsibilities was also evidence that continence care is undervalued and misunderstood, as it was deprioritised in favour of other work.

2549 ‘I feel that continence is not taken as a serious condition [by] the wider health service... we were the first department to be closed and are the only not to be fully re opened’

4265 ‘Continence Care is often seen as unimportant. This was evident in the pandemic’

Participants described the likely impact of diminished and delayed continence care, including increased pad costs, deconditioning of patient health and hospital admission. Perceptions that continence care was undervalued were further shared by participants who had not been redeployed, but who wanted the impact of good continence care to be better understood and championed amongst other teams and management in their organisations.

3531 ‘We want our managers and other health care professionals to see us as an important service …at times we don't always feel this’

#### Diminished influence in residential and care homes

Participants unable to access care/residential homes were distanced from patients and reliant on information from care staff to undertake assessments and make treatment plans. Diminished influence to promote strategies designed to maximise continence (in place of containment products) was also reported.

8186 ‘Not visiting residential homes - has reduced our influence in their continence promotion and the rapport we have historically had’

#### Benefits and disadvantages of remote working, training and education

Remote (video) provision had allowed education/training sessions for staff and/or patient groups (e.g., instruction for parents about toilet training) to continue, with potential to reach a larger audience due to convenience and lack of restrictions on attendance. However, interactions between participants and overall effectiveness of the sessions were perceived to be less when done remotely.

9792 ‘One to one sessions ... previously… in schools, these involve therapy-based work [are] less effective via video’

Although team meetings and supervisions could take place remotely, the ability of team members to support each other and have productive case discussions was limited by working remotely, and isolation was perceived as a risk.

4265 ‘Not able to see colleagues to offer support or discuss patients. It is not the same doing this online’

### Patient and carer response to community continence service changes

**Fig. 1:**
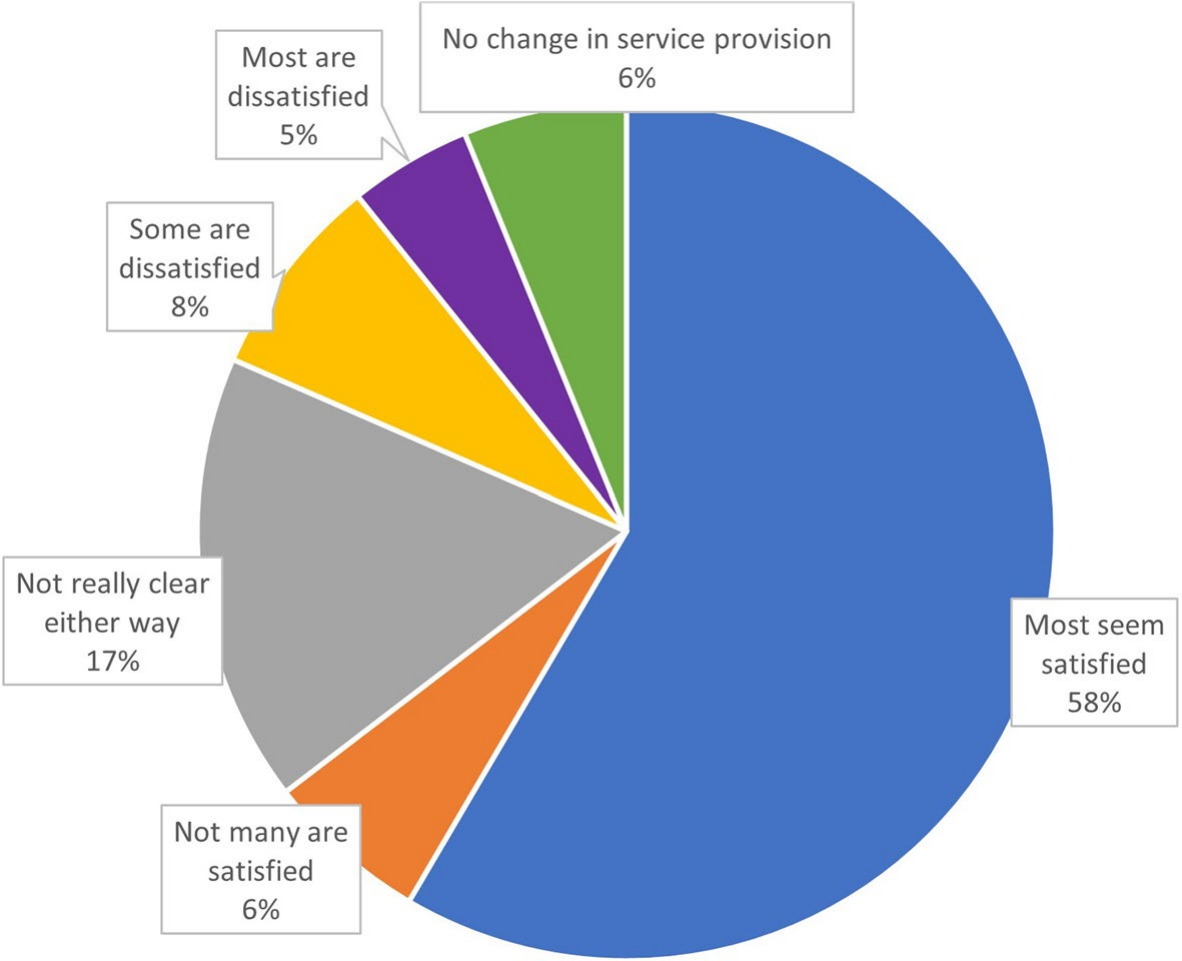
Survey Question 6 ‘What is your impression of the patient experience due to the changes in service provision?’

**Fig. 2:**
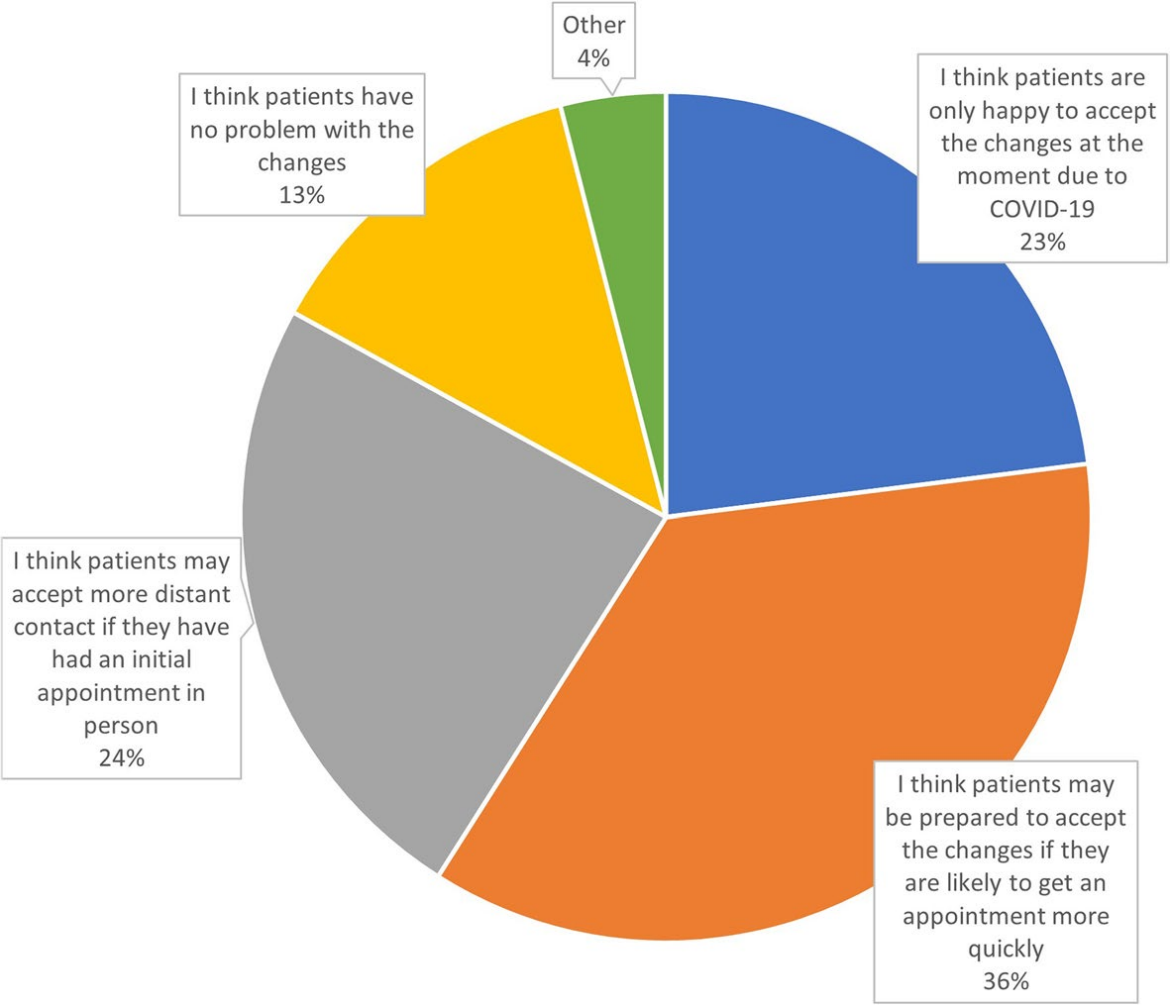
Survey Question 7 ‘Do you think patients may respond differently when we are beyond the COVID-19 situation’

Over half of participants perceived that all or most patients, carers and families had been ‘accepting’, or ‘understanding’ of changes (Q5, 5a), see also Figs. 1 and 2. Patient and carer gratitude that continence care and support had continued by remote means was commonly reported. However, complaints and frustration with long waiting lists were also reported.

### Future service provision

When asked about preferences for future continence care provision (Q12, Q13, Q14), participants overwhelmingly wanted to retain the greater flexibility for service delivery offered by use of remote appointments through continued (or increased) use of telephone/video consultations. However, return to in-person approaches was also desired for patient reassurance and relationship building, and for staff networking, clinical supervision and informal discussion opportunities, as well as training that may involve practical elements. Combined use of in-person and remote consultation options were perceived to offer a broadened and more flexible approach, which would be more accessible and expedite care for some patients.

Eleven participants intended to continue remote initial appointments, during which the professional would decide patient need for a subsequent ‘in-person’ appointment. In contrast, five participants emphasised the importance that initial assessments were undertaken in-person after which review appointments could be done remotely. Others favoured patient choice between telephone, video, or face-to-face appointments.

Participants wanted restoration of ‘the usual ways of working’ or implementation of planned service development/expansion that had ceased due to the pandemic; including return of redeployed staff; restoration of responsibilities to district/community nursing teams; end to personal protective equipment use; access to nursing homes and reinstated teaching and education opportunities. Freedom to choose between remote or in-person formats for team working, training and education was desired.

## Discussion

Community continence services have experienced major reorganisation in response to the pandemic, including redeployment of staff, additional case load and adoption of remote approaches to care delivery and team working, with consequent benefits and disadvantages for practice and care. Remote appointments prevent aspects of ‘usual’ assessment and have required professionals to decide patient need for examinations/tests from a distance. In-person appointments reduced, and services have varied in the criteria for which in-person appointments would be made. It is noteworthy that a proportion of participants intended to continue remote initial assessment of patients, where others favoured that initial assessment be done in-person. Guidance for the organisation of continence services mandates a physical assessment for all patients accessing continence care [19].

Remote appointments were perceived advantageous due to their efficiency, allowing more appointments in the time available, interventions to be started more rapidly, and greater convenience for patients evident in reduction in the number of patients that did not attend their appointment. However, remote approaches also had several disadvantages; assessments that were considered incomplete could impact waiting lists for in-person appointments; the lack of in-person interaction could potentially lead to missed diagnoses, inappropriate treatment or missed safeguarding opportunities because non-verbal cues and holistic signs were less likely to be evident; and the rapport and closeness between patient and professional was lessened. For some professionals remotely delivered care was potentially poorer quality care.

Further research is required to identify the interplay between service efficiency and patient convenience, and impact on care quality and outcomes for patients. In addition to potential consequences for patient care, remote working was perceived to affect relationships within continence teams by reducing opportunities for support and case discussion. The value and benefits of ‘in person’ connections were repeatedly emphasised for relationships and care delivery between professional and patient, and for supportive working within continence teams.

Over a third of participants reported staff redeployment and/or responsibility for additional case load at their service. The results of redeployment were largely negative, leading to increased waiting lists and minimal or delayed care for patients experiencing all but the most urgent continence problems. Those with additional case loads also reported potentially delayed care for continence patients, although some benefits were reported from closer working between continence and community nursing teams. Redeployment was perceived as evidence that continence care was poorly understood, deprioritised and increasingly diminished to pad provision, which had been exacerbated (though not originated) by the pandemic. Reduced access to and influence in care homes was also described as likely to diminish quality of care. These findings are concerning given the policy imperative and extensive evidence base for investment in high quality continence services [10, 19, 22].

### Relation to other studies

An assumption of improved care and outcomes for patients underlies a strong policy drive for increased use of technology in health and social care [6] of which virtual appointments are one element [30]. Previous studies have identified the convenience of telephone and video appointments and their advantages for some patients (younger/working patient groups) and those for whom accessing a clinic would be difficult [31, 32]. In common with previous studies, we have also identified limitations to the information available to professionals during remote consultation use, and consequent concerns about care quality [32, 33]. However, where an earlier study in primary care reported video consultations could overcome the lack of non-verbal cues characteristic of telephone consultations [31], participants in our study reported that non-verbal and holistic signs could be obscured during both telephone and video consultations; such that having ‘visual’ of the patient was not equivalent to being ‘in person’ with them.

While remote appointments may overcome difficulties of physical access, our study adds to findings of access and ease of use challenges associated with them [7], which necessitate careful consideration in relation to some patient groups. Groups perceived to be potentially disadvantaged were both adults and children with complex or safeguarding needs, older patient groups, those with hearing or visual difficulty and/or those without family support. Remote consultations also had the potential to undermine patient centred care if patients were unable to participate in the assessment due to young age, disability, or lack of ease with technology.

### Strengths and limitations

As far as we are aware this study is the first to report insights into the nature of changes made to community continence services in response to the COVID-19 pandemic and identify impacts on practice and care as experienced by frontline professionals. Opening the survey between Dec 2020 and June 2021 allowed participants to report experiences whilst services were still in a state of change/reorganisation and the impacts were being experienced day-to-day. Spending time identifying services, alongside direct and sometimes repeated contact with them, achieved a large and geographically broad sample of 65 participating services across at least 34 STP areas. Use of a survey design with open-ended questions allowed participants openness to report experiences unconstrained by narrowly defined response categories. This approach enhanced the richness of response data and benefitted the exploratory focus of the study. Dual coding of data, and thematic analysis of data vertically by question and horizontally across questions, with attention to inductively identified topics, increased reliability.

The size and breadth of our sample means findings are likely to be transferable to other settings and may contribute more generally to the corpus of research documenting the experiences within and impact on diverse NHS health services at this historically singular timepoint. The study has limitations: open-ended survey responses are not comparable in depth to responses given during in-depth interviews and cannot be extended or clarified through discussion between participant and researcher. Patients’ experiences of service changes require first-hand exploration [34]. We were also unable to invite all NHS community continence services to participate in the survey due to a lack of centrally held information about service locations across England.

### Implications for policy makers and clinicians

These findings can inform the design and delivery of future community continence services. 1) Redeployed services must be restored and staffed to provide high quality continence care with focus on achievement of continence over containment. 2) Remote appointments (telephone or video) offer considerable benefits. However, they should not be assumed to be accessible and convenient for all patient groups and should form one aspect in a flexible range of appointment options. 3) Research is required into the interplay between increased service capacity potentially offered by use of remote appointments and impact on patient care.

## Conclusion

Our study is unique in identifying the impact of changes made due to the COVID-19 pandemic on practice, accessibility and quality of care provided by both adult and children’s community continence services as perceived by continence professionals. We identify complex consequences linked to use of remote appointments (and remote working practices) with potential for both benefits and disadvantages for care quality, patient experience, and team working. Remote appointments offer efficiency and convenience. However, ‘in person’ interactions are highly valued for enabling optimum quality relationships, assessments and care between professional and patient. Failure to fully restore redeployed/diverted continence care staff, with sufficient resources to offer ‘in person’ appointments as appropriate to individual needs, will impart diminished health, social and quality of life burdens on patients, with associated costs to the NHS.

## Supplementary Information

**Additional file 1.** EPICCC-19 National Survey – questions and response rates. Includes wording of all open and closed-ended survey questions comprising the EPICCC-19 national survey. Includes total response rates for each question, and affirmative/negative response rates for questions which allow.

**Additional file 2.** Illustrative data extracts. Includes extracts of anonymised data taken from open ended survey questions that are illustrative of the themes described in the main text.

## Data Availability

The dataset(s) supporting the conclusions of this article is(are) included within the article (and its additional file(s)).

## Abbreviations

NHS: National Health Service
STP: Sustainability and Transformation Partnership
CCG: Clinical Commissioning Group
DNA: ‘Did not attend’ (appointment)
CN: Community nursing
DN: District nursing
