# Supplementary material for: Professional perspectives on impacts, benefits and disadvantages of changes made to community continence services during the COVID-19 pandemic: findings from the EPICCC-19 national survey
Source: BMC Health Serv Res. 2022 Jun 15;22:783. doi: 10.1186/s12913-022-08163-3 (PMC9199465; doi:10.1186/s12913-022-08163-3)
Supplement: Supplementary file 1 — Additional file 1. EPICCC-19 National Survey – questions and response rates. Includes wording of all open and closed-ended survey questions comprising the EPICCC-19 national survey. Includes total response rates for each question, and affirmative/negative response rates for questions which allow. [file 12913_2022_8163_MOESM1_ESM.docx]

**Additional file 1: EPICCC-19 National Survey Questions and response count**

| **Question Number** | **Question text** | **Per question response count (Total 65)** | **‘Affirmative/negative’ response count** |
| --- | --- | --- | --- |
| *Q1* | Consent process | 65/65 | - |
| Q2  2 a) | Please describe the nature of the continence service you provide (Tick box response)  Adult community service  Paediatric community service  Other  If you selected ‘Other’ please specify: | 65/65 | - |
| Q3 | Please tell us (in general) the geographical area covered by this service: | 60/65  (See Table 1) | - |
| Q4  Q4 a)  Q4 b) | How has the continence care that you provide changed in response to the COVID-19 situation? (please provide as much detail as you wish)  What are the benefits to these changes in your view, if any? (e.g., for patients, for continence professionals, for the service)  What are the drawbacks to these changes in your view, if any? (e.g., for patients, for continence professionals, for the service) | 65/65  64/65  64/65 | 64/65 reported changes  57/64 reported benefits. 6/64 reported no benefits.  61/64 reported drawbacks. |
| Q5  Q5 a) | How are patients responding to these changes?  How are carers / families responding to these changes? | 64/65  60/65 | 38/64 reported ‘positive’ response.  7/64 reported ‘mixed/negative’ response.  37/60 reported ‘positive’ response.  11/60 reported ‘negative’ response. |
| Q6 | What is your impression of the patient experience due to the changes in service provision? Tick box response (one option only):  Most seem satisfied  Not many are satisfied  Not really clear either way  Some are dissatisfied  Most are dissatisfied  No change in service provision | 65/65 | (See Fig 1) |
| Q7  Q7 a) | Do you think patients may respond differently when we are beyond the COVID-19 situation? Tick box response (multiple options allowed):  I think patients are only happy to accept the changes at the moment due to COVID-19  I think patients may be prepared to accept the changes if they are likely to get an appointment more quickly  I think patients may accept more distant contact if they have had an initial appointment in person  I think patients have no problem with the changes  Other  If Other, please provide as much information as possible to help us going forward: | 65/65 | (See Fig 2) |
| Q8 | Have you experienced any issues with patients not being able to access the services in their current format? (e.g., technology issues, people living with particular disabilities, other issues that prevent patients engaging with the current provision) | 63/65 | 39/63 reported access difficulties.  20/63 reported no access difficulties |
| Q9  Q9 a) | Do you think the changes in service provision offer an advantage for any groups/individuals? (Tick box response)  No  Yes  If Yes, please give detail of which groups and why: | 64/65 | 45/64 reported advantaged groups.  19/64 reported no advantaged groups |
| Q10  Q10 a) | Do you think any groups/individuals are disadvantaged by the changes in service provision? (Tick box response)  No  Yes  If Yes, please give details of which groups and why: | 65/65 | 43/65 reported disadvantaged groups.  22/65 reported no disadvantaged groups |
| Q11 | Are you aware of any impacts due to COVID-19 on either referral into, or referral out of your service? | 62/65 | 43/62 reported impacts. 19/62 reported no impacts |
| Q12 | Are you currently working in new ways that you would like to see continued and further developed post COVID-19? (e.g., telephone/video consultations) | 65/65 | 62/65 reported ways of working to continue using |
| Q13 | Are there elements of the current service that you would not like to see in place this time next year? | 62/65 | 29/62 reported ways of working to cease using |
| Q14  Q14 a)  Q14 b) | What would you like your service to look like in a year's time?  What would be the resulting impact for patients?  What would be the resulting impact on the service? | 64/65 | 64/65 reported preferences for future of their service |
| Q15 | What do you think the impact might be, if any, due to a delay in people accessing continence treatment due to the COVID-19 situation? | 62/65 | 54/62 reported potential impacts on patients  8/62 did not perceive delays at their service |
| Q16 | If you would like to add any further comments we would be very grateful to hear them. | 30/65 | - |
