# Supplementary material for: Professional perspectives on impacts, benefits and disadvantages of changes made to community continence services during the COVID-19 pandemic: findings from the EPICCC-19 national survey
Source: BMC Health Serv Res. 2022 Jun 15;22:783. doi: 10.1186/s12913-022-08163-3 (PMC9199465; doi:10.1186/s12913-022-08163-3)
Supplement: Supplementary file 2 — Additional file 2. Illustrative data extracts. Includes extracts of anonymised data taken from open ended survey questions that are illustrative of the themes described in the main text. [file 12913_2022_8163_MOESM2_ESM.docx]

**Additional file 2 – illustrative data extracts**

**Changes to adult and paediatric community continence services**

**From in-person to remote care provision:**

3361 ‘All initial appointments are now virtual and over the telephone with home visits arranged as deemed necessary’

6777 ‘Majority of patient assessment are being completed by teleclinics where previously patients were seen face to face. Any patients needing a bladder scan or visit are triaged by this process and booked a home visit. No clinics currently running at all’

1454 ‘We are now carrying out our 1st assessments via telephone, we only bring patients into the clinic if required’

2614 ‘Reduced face to face assessments, therefore unable to perform routine investigations such as skin integrity checks… bladder scan’

2984 ‘Face to face consultations have greatly reduced therefore basic continence assessments like urinalysis and bladder scanning were not done at the first consultation’

8186 ‘We don't get to scan everyone or test all urines routinely now - but we identify those that need it from our verbal assessment and arrange an appointment/send out urine sample equipment’

4895 ‘We changed our face to face assessments to telephone assessments, for all of the assessment excluding the physical aspects of the assessment’

3233 ‘…emergency and new patients are seen face to face once they are screened using a checklist via telephone’

9792 ‘Face to face appointments do run for those who we feel really need it, this can be due to bladder scanning, safeguarding issues or CYP who struggle with telephone or video calls'

7210 ‘[Only] bladder scanning ... continued face to face - covid secure

2113 ‘We restored in [month]... to again be suspended ... for a further 3 months’

1194 ‘We paused new patient appointments for a time’

3361 ‘Since [month] face to face clinics have resumed for reviews of continence care, examination and scans’

2455 ‘Consultations are now non face to face’

**Redeployment, service suspension, additional responsibilities:**

0896 ‘We were redeployed, those at home shielding continued to triage and telephone assess patients for containment [and would] contact those redeployed [when] verbal assessment suggested [patient’s] symptoms needed action now [and could not be dealt with remotely]’

9422 ‘Two members of staff are working from home carrying out assessments ... [by] telephone. Home visits for urgent cases are carried out by redeployed staff. There are no treatment clinics’

3755 ‘[We] continued with a reduced service, virtual appointments, [a percentage] of these are advice and to hold until physical appt can be resumed’

2162 ‘Patients with Specialist Nurse needs are being placed on a waiting list as Specialist Nurses have been redeployed’

8821 ‘Our adult case load was closed for 3 months … and we were not allowed to accept referrals. Therefore we worked and supported community teams with catheter care and continence assessments’

6777 ‘The service is also supporting the community nursing teams at times of pressure with catheterisations and [Trial without catheter] when capacity allows’

5562 ‘Community nursing patients are usually assessed whilst being visited for other care plans and now they are being passed onto the continence service’

**Access to residential and care homes:**

5163 ‘We are still seeing patients in clinics, at home [visits] and if able to gain access into the nursing homes’

9053 ‘Not able to review and assess in residential and nursing homes due to them being closed to professionals’

4588 ‘Initial assessment done by phone with carers - clinician decides if H[ome] V[isit] for physical examination required to reduce care home footfall.’

P1 ‘Not allowed to visit the care homes (by their request)’

**Remote working, training and educational activities**:

2113 ‘[Microsoft] teams for meetings’

2614 ‘Some video conferencing delivery of training’

4265 ‘Remote training via Teams carried out’

2614 ‘Some delay in educational competencies being completed due [number] of candidates allowed in one room’

4856 ‘Reduced training sessions and candidate numbers’

8186 ‘All clinical training stopped’

**Perceived benefits and disadvantages of changes to community continence services**

**Remote care provision - efficiencies and limitations:**

Efficiencies: 2455 ‘Shorter contacts improving capacity for the service, able to access the children sooner from point of referral’

2614 ‘Reduce[d] waiting list time for clinic appointments as initial assessments were commenced via telephone consultations’

4895 ‘Easier access to the Continence team, over the phone, reduced waiting times and earlier health advice and intervention’

Incompleteness: 7749 ‘We have not given proper assessment as we would as patient need face to face assessment’

4840 ‘We were not able to provide the usual care i.e. physical examinations at the initial appointment’

8603 ‘[telephone clinic] does not allow for examination and therefore the consultation is not complete’

0402 ‘Not being able to examine child, particularly for faecal impaction in children who are soiling’

7663 ‘Not being able to complete an assessment at the time of initial contact can be concerning especially when chronic urinary retention may be a possibility’

2614 ‘Potential increase in moisture lesions/skin integrity compromised as unable to do face to face assessments’

9053 ‘Not able to assess pressure area skin care’

3233 ‘Some diagnosis are missed hence leading to lack of appropriate treatment plan’

2614 ‘Potential for inappropriate products being prescribed [due to] not being able to complete initial face to face assessments’

Less effective: 8186 ‘We thought telephone appointments would be quicker - we can't do all the practical things and we don't have the travel between community visits, but actually they can take longer as we can't see what is happening in the home and are relying on information, that can be selective’

1194 ‘Unable to observe body language when asking questions particularly if safeguarding issues’

5163 ‘Some patients engage less over the phone and often do not understand all of the information given, the staff member would not always pick up all issues if they do not see the patient face to face’

9053 ‘Relying on the information from care staff regarding patients symptoms and formulating a diagnosis and plan of care from this information’

0402 ‘[I] explain to [parent] how to feel their child's abdomen to check for faecal impaction’

1454 ‘Less confident we are giving the best to our patients who struggle to use the phone’

6777 ‘Nurse/patient relationship altered by telephone appointments for a very personal assessment such as continence’

3531 ‘Drawbacks are that we haven't been able to have the rapport with our patient’

**Remote care provision - convenience and inaccessibility:**

Convenience: 9053 ‘Reduced DNA rate for Adults and Paeds service’

8012 ‘Some parents find the financial cost of public transport prevents them from accessing clinic appointments’

1786 ‘Patients are liking that they do not have to travel to a clinic venue’

7663 ‘Patients have been able to get support and treatment despite not wishing to attend a clinic and are not having to travel to be seen’

3355 ‘Elderly patients who find it difficult to attend clinic appointments by themselves would benefit from telephone consultations’

4281 ‘Easier conversations with young people over the telephone as they are less shy etc to talk’

Access challenges: 3233 ‘Some of our patients find it difficult to hold conversation on the phone mainly due to language barrier, vulnerable patients like [those with] learning disabilities, mental health [challenges] find [engaging difficult]’

2990 ‘We have very few patients that would be able to participate in video consultations due to mainly elderly and do not have computer or unable cognitively to be able to’

7663 ‘Patients with communication or cognitive difficulties can be at a disadvantage especially if they do not have anyone to speak for them’

7210 ‘Loss of the child's voice/engagement of the child as often parent on the phone’

2162 ‘Telephone consultations has meant assessment not always includ[ing] patient themselves’

8603 ‘For some individuals [telephone consultations] has been negative (e.g. adolescents can't personally engage)’

**Impact of redeployment and additional responsibilities:**

2162 ‘Patients who require Specialist Nurse input are not receiving any service during lockdown due to redeployment’

3416 ‘We have been partially shut down twice now and we have an ever-growing waiting list as a result’

8186 ‘We expect it to take up to a year before we are seeing clinic patients in our 'pre-covid' time frames - of within 6 weeks of referral’

5562 ‘The continence service is serving their own waiting list and the community nurse’s as they have less capacity and are required to visit the priority patients before continence’

7450 ‘Housebound patients not getting assessed as this is normally done by Community Nurses’

1549 ‘Took over [other service] workload, and maintained our own work load by starting early and finishing later – no overtime paid’

4588 ‘Stress and anxiety of knowing we have patients waiting so long for support’

0896 ‘Greater understanding how our neighbourhood teams work’

6777 ‘Reskill of continence staff to catheters etc with community nurse work’

**Continence care downgraded and undervalued:**

7663 ‘Patients have potentially been given incontinence pads quicker than they might have pre-covid’

6543 ‘[service] has been de prioritised and become a pad supply service...no effort towards trying to gain continence’

4265 ‘Whilst it is essential that insulins and dressings etc are done. Continence assessments if not carried out in a timely manner can also have a devastating effect on the sufferer and their family and lead to hospital admissions’

9053 ‘... bladder and bowel teams are seen as pads services and need to have their profile raised to show how skilled they are and the impact we have on patients’

3531 ‘We want continence care/nursing to be seen has an important part of the NHS. At times it is seen has not important has it is not life threatening but it is life debilitating’

**Diminished influence in residential and care homes**:

9053 ‘Nursing and Residential Home are focused on containment products only and not other life style changes and interventions’

4588 ‘Residential care homes - getting them to send in completed bladder and bowel diaries we would get one or the other - so some admin chasing’

**Benefits and disadvantages of remote working, training and education:**

1194 ‘Lack of parent-to-parent interaction during training sessions’

1194 ‘Peer supervision has continued via MS Teams however we miss the ad hoc case discussions and the face-to-face regular contact with colleagues’

4281 ‘A particular challenge is supporting each other within the team and encouraging self-care…working remotely can be isolating for some, yet motivating for others’

3361 ‘Staff also need to be able to get together, network have clinical supervision and a chat, it helps us all reduce loneliness and isolation. Training also needs to be face-to-face esp. with the practical elements’
